# Factors Associated with the Efficiency of Home Non-Invasive Ventilation in Patients with Obesity-Hypoventilation Syndrome in Martinique

**DOI:** 10.3390/jcm12103381

**Published:** 2023-05-10

**Authors:** Moustapha Agossou, Ramona Barzu, Bérénice Awanou, Joelle Bellegarde-Joachim, Jean-Michel Arnal, Moustapha Dramé

**Affiliations:** 1 Department of Respiratory Medicine, CHU of Martinique, 97261 Fort-de-France, France; 2 Réseau Respi-R, DAC Martinique, 97200 Fort-de-France, France; 3 Service de Réanimation Polyvalente, Hôpital Sainte Musse, 83100 Toulon, France; 4 Department of Clinical Research and Innovation, CHU of Martinique, 97261 Fort-de-France, France; 5 EpiCliV Research Unit, Faculty of Medicine, University of the French West Indies, 97261 Fort-de-France, France

**Keywords:** obesity-hypoventilation syndrome, comorbidity, non-invasive ventilation, persistent hypercapnia, home ventilation

## Abstract

Obesity-hypoventilation syndrome (OHS) is a respiratory complication of obesity characterized by chronic hypercapnic respiratory failure. It is often associated with several comorbidities and is treated by positive airway pressure (PAP) therapy. This study aimed to identify factors associated with persistent hypercapnia in patients receiving home non-invasive ventilation (NIV). We performed a retrospective study including patients with documented OHS. In total, 143 patients were included (79.7% women, age 67 ± 15.5 years, body mass index 41.6 ± 8.3 kg/m^2^). After 4.6 ± 4.0 years of follow-up, 72 patients (50.3%) remained hypercapnic. In bivariable analysis, clinical data showed no difference in follow-up duration, number of comorbidities, comorbidities, or circumstance of discovery. Patients with persistent hypercapnia on NIV were generally older, with lower BMI and more comorbidities. (5.5 ± 1.8 versus 4.4 ± 2.1, *p* = 0.001), female sex (87.5% versus 71.8%), was treated by NIV (100% versus 90.1%, *p* < 0.01), had lower FVC (56.7 ± 17.2 versus 63.6 ± 18% of theoretical value, *p* = 0.04), lower TLC (69.1 ± 15.3 versus 74.5 ± 14.6% of theoretical value, *p* = 0.07), lower RV (88.4 ± 27.1 versus 102.5 ± 29.4% of theoretical value, *p* = 0.02), higher pCO2 at diagnosis (59.7 ± 11.7 versus 54.6 ± 10.1 mmHg, *p* = 0.01) and lower pH (7.38 ± 0.03 versus 7.40 ± 0.04, *p* = 0.007), higher pressure support (12.6 ± 2.6 versus 11.5 ± 2.4 cmH_2_O, *p* = 0.04) and lower EPAP (8.2 ± 1.9 versus 9 ± 2.0 cmH_2_O, *p* = 0.06). There was no difference in non-intentional leaks and daily use between patients between both groups. By multivariable analysis, sex, BMI, pCO2 at diagnosis, and TLC were independent risk factors for persistent hypercapnia on home NIV. In individuals with OHS, persistent hypercapnia on home NIV therapy is frequent. Sex, BMI, pCO2 at diagnosis, and TLC were all associated with an increased risk of persistent hypercapnia in persons treated with home NIV.

## 1. Key Messages

Obesity hypoventilation syndrome is associated with many comorbidities, including cardiovascular and metabolic. Persistent hypercapnia is frequent and deleterious for patients. There is a paucity of data on the factors associated with persistent hypercapnia on home NIV after optimizing PAP settings. Sex, BMI, pCO2 at diagnosis, and TLC are independently associated with persistent hypercapnia on home NIV.

## 2. Introduction

Obesity is a major public health problem worldwide and its prevalence has increased significantly in recent years [1]. Obesity is associated with cardiovascular, metabolic, neoplastic, respiratory, mechanical, and other complications [1,2]. Obesity also leads to impaired respiratory function [3], which has consequences such as obstructive sleep apnea syndrome (OSA), obesity-hypoventilation syndrome (OHS), asthma, and chronic obstructive pulmonary disease (COPD) [4]. OHS is a type of chronic hypercapnic respiratory failure associated with several comorbidities, including cardiovascular and metabolic complications [5]. The physiopathology of OHS is related to 3 key mechanisms [3], namely an obesity-related change to the respiratory system with the creation of pulmonary restrictive syndrome; an alteration of respiratory drive, especially in rapid eye movement (REM) sleep; and breathing abnormalities during sleep with OSA in obese patients.

Martinique is a French department of the West Indies. The Caribbean population is predominantly composed of French citizens of Afro-Caribbean origin. The prevalence of obesity, diabetes, and arterial hypertension is higher in this population than in metropolitan France [6,7]. Obesity is a serious problem in Martinique, with 33% of the population overweight, and 20% obese [8], most of them women [9]. On the other hand, tobacco consumption is low, and just 16% of the population are smokers, which is only half the proportion observed in metropolitan France [10]. OHS is the leading cause of chronic hypercapnic respiratory failure. The management of OHS is multidisciplinary, combining nocturnal ventilatory support with the management of obesity and comorbidities [11]. Nocturnal ventilatory treatment [11] uses continuous positive airway pressure (CPAP) or bi-level positive airway pressure (BIPAP). Even with nocturnal non-invasive ventilation (NIV), some patients still have persistent hypercapnia [12], which may be responsible for considerable morbidity and mortality [13,14].

To the best of our knowledge, no study has evaluated the impact of comorbidities on the course of OHS and the factors that may affect outcomes. The objective of this study was therefore to describe the profiles of OHS patients in Martinique and to assess the factors associated with persistent hypercapnia in patients receiving nocturnal positive airways pressure (PAP) therapy at home.

## 3. Patients and Methods

We performed an observational, single-centre, retrospective study in the Department of Respiratory Medicine of the University Hospital (CHU) of Martinique, which is the referral centre for patients with respiratory diseases for the whole island, and where all patients with chronic respiratory failure, including OHS, are treated.

### 3.1. Eligibility Criteria

Patients were included from 1 January 2019 to 31 December 2022. All patients followed in our Department for OHS and hospitalized in the department during the study period were eligible. The inclusion criteria were:

Age 18 years or older

Diagnosis of OHS, including body mass index (BMI) ≥ 30 Kg/m^2^, daytime partial pressure of carbon dioxide (PCO2) ≥ 45 mmHg, and absence of COPD, neuromuscular disease, or hypothyroidism.

We excluded patients with a smoking history of more than 10 pack-years in women or 15 pack-years in men, as this may be associated with obstructive syndrome where associated COPD cannot be ruled out.

### 3.2. Patient Follow-Up

Patients had regular follow-ups every 6 to 12 months in our Department to optimize NIV settings and monitor comorbidities. In follow-up, patients were admitted for 3 to 5 days, to optimize the device settings for the home NIV device, and to monitor comorbidities. The objective is to correct hypercapnia by reaching an optimal balance between the increase in pressure support leaks, apnea-hypopnea index (AHI), and monitoring ventilator asynchrony.

### 3.3. Initiation of NIV

BIPAP was initiated during hospitalisation. Most patients being followed-up were diagnosed in the context of acute decompensation, and BIPAP was initiated at that time. For patients already receiving CPAP, expiratory positive airway pressure (EPAP) was set at a level equal to CPAP minus 2 cm H_2_0. Inspiratory assistance was then programmed to achieve the optimal balance between efficiency and leaks. For patients who were not receiving CPAP, parameters were set empirically, by adjusting according to residual apnea-hypopnea index (AHI), asynchrony, leaks, and patient complaints.

### 3.4. Comorbidities

Comorbidities were assessed based on the biological work-up and the patient’s ongoing treatment. If there were any doubts, a relevant specialist was consulted to confirm or rule out the diagnosis. Cardiovascular comorbidities were diagnosed based on electrocardiographic and/or echocardiographic findings, and a cardiologist’s opinion. Respiratory comorbidities, notably asthma, were assessed based on clinical symptoms, obstructive ventilatory disorders, and absence of heavy smoking or professional exposures. Patients all underwent polygraphy or polysomnography, either before diagnosis and were already receiving CPAP for severe obstructive sleep apnea, or at the time of diagnosis. Severe obstructive sleep apnea was defined as an AHI > 30/hour.

### 3.5. Data Recorded

We recorded sociodemographic data (age, age at diagnosis, BMI), comorbidities at diagnosis, comorbidities at inclusion in the cohort, treatment initiated (CPAP, NIV, NIV combined with oxygen therapy), and follow-up data on persistent hypercapnia for all patients.

Persistent hypercapnia was defined as a diurnal PaCO2 > 45 mmHg (6 KPa) in room air without acute on chronic respiratory failure. We considered the value of arterial gas, as well as treatment data (ventilator settings, interface, leaks, and daily use) during the last routine visit without exacerbation.

### 3.6. Statistical Analysis

A descriptive analysis was performed. Quantitative variables are described as mean ± standard deviation (SD), and we compared them using the Student *t*-test. Categorical variables are described as numbers and percentages and were compared using Fisher’s exact test. Bivariable and multivariable analyses for the associations between covariates and the persistence of hypercapnia were performed using binary logistic regression modelling. Statistical analyses were performed using SAS version 9.4 (SAS Institute Inc., Cary, NC, USA). Tests were considered significant for *p*-values < 0.05.

### 3.7. Ethical Considerations

The study was performed in accordance with the Declaration of Helsinki and was approved by the Institutional Review Board of the University Hospitals of Martinique under the number 2020/073. At each follow-up consultation or hospitalization, patients received an information leaflet explaining that their routine medical data could be used for research purposes, and indicating how they could explicitly oppose this use, if they so desired, in accordance with French legislation governing retrospective studies using routine medical data. No patient explicitly opposed the use of their medical data for the purposes of the present study.

## 4. Results

One hundred and forty-three patients were included, of whom 114 (79.7%) were women. The patients included were diagnosed between 5 October 2001 and 30 September 2022. The mean age at diagnosis was 67.3 ± 15.5 years, and the mean age at inclusion was 71.7 ± 15.2 years. The mean BMI was 41.6 ± 8.3 kg/m^2^.

After a mean follow-up period of 4.58 ± 4.04 years, 72 patients (50.3%) had persistent hypercapnia, despite optimal settings on their home NIV device and management of comorbidities. The characteristics of the study population are displayed in Table 1, and compared between those with and those without persistent hypercapnia. In bivariate analysis, there was a significant difference between groups in terms of sex, age, BMI, Charlson comorbidities index, and modality of home positive airway pressure device. Spirometry and blood gas data from the study population are shown in Table 2. In bivariate analysis, there was a significant association between each FVC and residual volume, and the persistence of hypercapnia. On blood gas analysis at diagnosis, only pCO2 was found to be significantly higher in patients with persistent hypercapnia. At the time of follow-up, when patients were included in the study, all the blood gas parameters were found to be significantly different between groups.

All patients with BIPAP had persistent hypercapnia, while none of the patients receiving CPAP had persistent hypercapnia. There was no difference in the length of daily use, or unintentional leaks, between patients with and without persistent hypercapnia, but in ventilator settings, the pressure support was higher in the group with persistent hypercapnia (Table 3).

By multivariate analysis, sex, BMI, pCO2 at diagnosis, and total lung capacity were found to be significantly associated with the risk of persistent hypercapnia (Table 4).

## 5. Discussion

The most frequent comorbidities in our population were arterial hypertension, diabetes mellitus, hypercholesterolemia, chronic cardiac failure, and asthma. After a follow-up period of 4.8 ± 4 years, 50.3% of patients still had PaCO2 > 45 mmHg (6 kPa). Sex, BMI, pCO2 at diagnosis, and TLC were found to be independently associated with the risk of persistent hypercapnia. An increase of 1 kg/m^2^ of BMI decreased the risk of persistent hypercapnia by 8%, a 1% increase in TLC decreased the risk by 4%, an increase in pCO2 at diagnosis of 1 mmHg increased the risk by 5%, and being a man decreased the risk by 75%. Daily use of NIV, non-intentional leaks, and settings were similar in both groups, but patients with persistent hypercapnia had higher pressure support. Surprisingly, BMI seems to have a protective effect against persistent hypercapnia. The restrictive syndrome, measured by total lung capacity, increases the risk of persistent hypercapnia, as does pCO2 at the time of diagnosis.

The mechanisms of diurnal hypercapnia in OHS are complex, and include hypoventilation during nighttime sleep and especially, persisting during the daytime [15,16]. There is also a reduced ventilatory response to hypercapnia [16] and overproduction of carbon dioxide that is not compensated for by an increase in alveolar ventilation [17].

In our study, patients who had persistent hypercapnia were less obese but had great restrictive syndrome. This restrictive syndrome seems to have been the determinant feature driving the persisting hypercapnia, but this result is likely influenced by several specificities unique to our population.

Firstly, the subjects in our study were, in general, older, with an average age at diagnosis was 67.3 ± 15.5 years, and 71.7 ± 15.2 at the time of inclusion. This is higher than reported elsewhere; for example, BaHammam et al. reported a mean age of 61.5 ± 11.9 years in women, and 49.1 ± 12.5 in men [18]. Maza et al. and Mokhlesi et al. both reported younger mean ages in their cohorts [19,20]. In bivariate analysis, there was a significant difference in age between those with vs. those without persistent hypercapnia, although it was no longer significant in multivariate analysis.

Second, regarding comorbidities, there were high rates of certain comorbidities in our study, including asthma and chronic heart failure. Asthma is a chronic pulmonary disease with a higher prevalence in the French West Indies compared to mainland France [21,22]. Borel et al. reported only 13% of asthma in OHS patients in their cohort [23]. Obese asthma is known for its moderate reversibility [24] and can mimic chronic obstructive pulmonary disease (COPD) albeit with more difficulty normalizing arterial blood gas and requiring high-pressure support [25,26]. Our population of African origin is particularly afflicted by cardiovascular diseases [27] and OSA [28].

There is also the question of the possible role of pulmonary resistance and compliance in response to NIV. Further prospective studies with adequately powered sample sizes are warranted to elucidate the particularity of OHS in black people, the exact role between obesity, restrictive syndrome, and persisting hypercapnia

Regarding the role of sex, a few studies found a female predominance, as in our study [18,29,30], but no study to date has specifically investigated the impact of sex on the progression of the disease. In a previous study, we reported this female predominance in OHS [31].

Regarding age, it was not found to be an independent factor associated with persistent hypercapnia in our analysis.

Normalization of PaCO2 after initiation of nocturnal home ventilation varies between 2% and 74% [12]. Soghier et al. reported in a meta-analysis that hypercapnia resolved in 46.6% at 3 months, and 51.9% at 3 years [32]. Chronic hypercapnia is associated with an increased risk of morbidity and mortality in patients with OHS [13,14], hence the need to aim for an improvement in the blood gas results of these patients.

There is a paucity of data in the literature regarding the factors potentially associated with persistent hypercapnia over the long-term. Mokhlesi et al. identified variables that predicted changes in PaCO2 after PAP therapy and found that average daily hours of positive pressure therapy, FEV1 percentage of predicted, and baseline PaCO2 were all significant modifiable predictors of improvement in pCO2 [20]. Howard et al. reported that baseline PaCO2 predicted persistent ventilatory failure on treatment [33]. Javaheri et al. further reported that body weight is a determinant of daytime hypercapnia in OHS, but also underlined the extent of the functional respiratory anomalies. At similar BMI, patients with restrictive or obstruction syndrome were the most hypercapnic [16]. Persistent hypercapnia in the long term does not appear to depend either on adherence to therapy (with no significant difference), or on support pressure (non-significantly higher in the hypercapnic group in our study). Indeed, it has been shown that 4.5 h of daily use achieves improvements in hypercapnia and hypoxia, and benefits plateau after about 7 h of use [20].

We expected that the severity of obesity would be associated with the restriction, but paradoxically, very obese patients with less restriction are probably easier to ventilate than patients who are less obese but with greater restriction. The mechanisms of OHS are extremely complex and do not solely depend on obesity, but also on its impact on respiratory function [34]. The specificity of our population, in addition to the mainly African origin, is that they were older, with more comorbidities, such as heart failure and asthma, which each affected around one-quarter of the patients. There may also be an effect of race at play in the observation that those with lower BMI had a higher likelihood of OHS. It is known that individuals of East Asian origin develop obstructive sleep apnea at lower BMI levels than their non-Asian counterparts, due to cephalometric differences [35], and thus, OHS may also be more prevalent at correspondingly lower BMI values. Possible craniofacial anatomic features influencing OSA include mandibular deficiency, tongue and soft palate enlargement, and inferior displacement of the hyoid bone. Balachandran et al. reported that the average BMI of 291 OHS patients from 4 studies among Japanese subjects was 32 kg/m^2^, vs a mean of 44 kg/m^2^ among 757 OHS patients from 10 studies involving mostly non-Asian subjects [36]. Of note, in our study, although there was a significantly higher likelihood of OHS in patients with lower BMI, the average BMI levels were 43 and 40 kg/m^2^ in the two groups, which is in line with the average values reported for non-Asian subjects. Nevertheless, there may be other factors at play. Although the differences did not reach statistical significance, the subjects with persistent hypercapnia were older and also had numerically higher rates of arterial hypertension, diabetes, and a lower rate of pulmonary hypertension. There may therefore be macro- and microvascular mechanisms involved that cannot be elucidated by the present data, and warrant further investigation, in addition to the effect of ethnic origin.

To the best of our knowledge, few previous studies have evaluated the factors associated with persistent alveolar hypoventilation in patients on nocturnal home NIV for OHS after optimization of the device settings. Similarly, no study has been performed on this subject in the Martinican population, a population comprising predominantly persons of African descent, with their inherent specificities, and indeed, who are often excluded from studies on the subject. This population has a higher prevalence of obesity, diabetes, and arterial hypertension than the population of metropolitan France.

The strengths of this study include the follow-up of patients in the same center, which ensures consistent follow-up methods. However, limitations include the fact that this was a retrospective, single-centre study with all the related shortcomings, including reduced statistical power. Data are missing for certain variables, particularly those regarding the BMI of some obese and bedridden patients that our center is not able to weigh. Further studies are warranted to elucidate the relationship between factors such as age, sex or BMI, and persistent hypercapnia.

## 6. Conclusions

OHS is a complication of obesity that affects mainly women and the elderly in the Martinican population. It is associated with many comorbidities such as cardiovascular diseases, metabolic diseases, and asthma. Persistent hypercapnia under NIV is present in 50.3% of patients. Female sex, lower BMI, total lung capacity, and high baseline pCO2 were all independently associated with persistent hypercapnia under home NIV. Knowing the independent factors that may positively impact blood gas results could help to guide measures to optimize the management of these factors for better follow-up. Special attention should be paid to these factors in the follow-up of these patients. Further studies are warranted to further elucidate the relationship between these factors and persistent hypercapnia. 

## Figures and Tables

**Table 1:** Patient characteristics.

Characteristics	*n*	%	pCO2 ≤ 45 mmHg *n* = 71	pCO2 > 45 mmHg *n* = 72	*p*
Female sex	143		51 (71.8)	63 (87.5)	0.04
Age at diagnosis	143		62.5 ± 15.3	71.8 ± 14.4	<0.001
Age at inclusion	143		66.9 ± 14.3	76.4 ± 14.6	<0.001
Follow up duration	143		4.5 ± 4	4.7 ± 4.1	0.8
Body mass index	128		43.2 ± 8.7	40 ± 7.4	0.03
Number of comorbidities	142		3.7 ± 1.5	3.8 ± 1.5	0.7
Charlson index	142		4.4 ± 2.1	5.5 ± 1.8	0.001
Comorbidities *N* = 143					
Age ≥ 70 years at diagnosis	69	48	21	48	<0.0001
Age ≥ 70 years at inclusion	85	59	31 (43.7)	54 (74)	<0.001
Arterial hypertension	121	84	57 (80.3)	64 (87.7)	0.3
Severe obstructive sleep apnea	103	71.5	56 (78.9)	47 (64.4)	0.07
Diabetes mellitus	85	59	36 (50.7)	49 (67.1)	0.06
Dyslipidemia	40	27.8	19 (26.8)	21 (28.8)	0.9
Asthma	35	24.3	18 (25.4)	17 (23.3)	0.8
Cardiac arrhythmia	28	19.4	13 (18.3)	15 (20.6)	0.8
Chronic heart failure	35	24.3	17 (23.9)	18 (24.7)	1
Pulmonary hypertension	17	11.8	10 (14.1)	7 (9.6)	0.4
Lower limb arteriopathy	12	8.3	5 (7)	7 (9.6)	0.8
Circumstances of discovery of the disease *N* = 143					
Acute respiratory failure	109	75.7	52 (73.2)	57 (78.1)	0.6
Follow-up for sleep apnea	26	18.1	13 (18.3)	13 (17.8)	1
Treatment					
BIPAP	136	95.1	64 (90.1)	72 (100)	0.01
BIPAP with oxygen	12	08.3	6 (8.5)	6 (8.2)	1
CPAP	07	04.9	7 (100)	0	0.006

pCO2: partial pressure of carbon dioxide, pO2: partial pressure of Oxygen, sd: standard deviation, BIPAP: Bilevel positive airway pressure, CPAP: continuous positive airway pressure, *n* = number of subjects, and *N* = total number of subjects.

**Table 2:** Spirometry and blood gas data from the study population.

Parameters		pCO2 ≤ 45 mmHg *n* = 58	pCO2 > 45 mmHg *n* = 51	*p*
Spirometry (mean ± sd)	FEV1 (%)	59.7 ± 17.9	55.6 ± 17.4	0.2
	FVC (%)	63.6 ± 18	56.7 ± 17.2	0.04
	FEV1/FVC	77.5 ± 12	79.9 ± 15.2	0.4
	TLC (%)	74.5 ± 14.6	69.1 ± 15.3	0.07
	RV (%)	102.5 ± 29.4	88.4 ± 27.1	0.02
	ERV (%)	62.9 ± 39	75 ± 56.4	0.4
Blood gas at diagnosis (mean ± sd)	pH	7.36 ± 0.06	7.34 ± 0.06	0.2
	PaO2	66.9 ± 13.8	64.7 ± 13.5	0.4
	PaCO2	54.6 ± 10.1	59.7 ± 11.7	0.01
	Bicarbonate	30.2 ± 5.3	31.5 ± 5.2	0.2
Blood gas at follow-up (mean ± sd)	pH	7.40 ± 0.04	7.38 ± 0.03	0.007
	PaO2	77.8 ± 12.1	69.9 ± 12.2	<0.001
	PaCO2	41.7 ± 3.4	49.9 ± 4.6	<0.0001
	Bicarbonate	25 ± 2.1	29 ± 4	<0.0001

pCO2: partial pressure of carbon dioxide, FEV, forced expiratory volume; FVC, forced vital capacity; TLC, total lung capacity; RV, residual volume; ERV, expiratory reserve volume; pO2: partial pressure of Oxygen, sd: standard deviation.

**Table 3:** NIV settings, unintentional leaks, and daily use.

	All Patients *n* = 92	PaCO2 ≤ 45 mmHg *n* = 44	PaCO2 > 45 mmHg *n* = 48	*p*
Daily use (hours)	5.8 ± 3.0	6.3 ± 3.2	5.5 ± 2.2	0.2
IPAP (cmH_2_O)	20.7 ± 3.2	20.4 ± 3.2	21.0 ± 3.1	0.4
EPAP (cmH_2_O)	8.6 ± 2.0	9 ± 2.0	8.2 ± 1.9	0.06
Pressure support (cmH_2_O)	12.1 ± 2.5	11.5 ± 2.4	12.6 ± 2.6	0.04
Face mask/nasal mask (%)	78/22	74/26	83/17	NS
Unintentional leaks (L/min)	8.0 ± 14.0	7.0 ± 9.0	9.0 ± 18.0	0.546

IPAP: Inspiratory positive airway pressure, EPAP: expiratory positive airway pressure.

**Table 4:** Factors independently associated with persistent hypercapnia by multivariable analyses.

	OR	95% CI	*p*
Age at diagnosis ≥ 70 years	2.37	0.87–6.48	0.09
Male Sex	0.25	0.07–0.88	0.03
pCO2 at diagnosis	1.05	1.01–1.10	0.04
Body mass index	0.92	0.85–0.98	0.02
Total lung capacity	0.96	0.93–0.99	0.02

OR: Odds Ratio, CI: Confidence Interval, pCO2: partial pressure of carbon dioxide.

## Data Availability

The datasets generated and/or analyzed during the current study are available from the corresponding author on reasonable request.

## Abbreviations

BIPAP	Bi-level positive airway pressure
BMI	body mass index
CHU	university hospital center
COPD	chronic obstructive pulmonary disease
CPAP	continuous positive airways pressure
DAC	support and accompaniment system of Martinique
EPAP	expiratory positive airway pressure
ERV	Expiratory reserve volume
FEV1	Forced expiratory volume in 1 s
FVC	Forced vital capacity
IPAP	inspiratory positive airway pressure
KPa	kilopascal
mmHg	millimeter of mercury
NIV	non-invasive ventilation
NS	non-significant
OSA	obstructive sleep apnea
OHS	obesity-hypoventilation syndrome
PaCO2	partial pressure of carbon dioxide
PaO2	partial pressure of oxygen
PS	pressure support
RV	Residual volume
TLC	Total lung capacity
